# Cardiac Contractility Modulation Improves Left Ventricular Function, Including Global Longitudinal Strain, in Patients with Chronic Heart Failure

**DOI:** 10.3390/jcm14072251

**Published:** 2025-03-26

**Authors:** Cornelia Raab, Peter Roehl, Matthias Wiora, Henning Ebelt

**Affiliations:** Department of Medicine II, Catholic Hospital “St. Johann Nepomuk”, Haarbergstr. 72, 99097 Erfurt, Germany

**Keywords:** cardiac contractility modulation, left ventricular strain, heart failure

## Abstract

**Background**: Cardiac contractility modulation (CCM) is a therapy for patients with chronic heart failure with reduced ejection fraction (HFrEF). However, so far, there is no data available as to whether the application of CCM leads to changes in left ventricular global strain (GLS). This might be of special interest because GLS is known to be a strong predictor of outcomes in patients with HFrEF. **Methods**: Patients over 18 years old with heart failure with impaired left ventricular function (LVEF < 45%), a QRS complex < 130 ms, and NYHA classes II–IV despite guideline-directed medical therapy who planned to receive CCM implantation within 6 months were prospectively included into this study. Every 3 months, the status regarding CCM therapy was determined, and a standardized echocardiographic examination including the determination of LV global longitudinal strain was performed. **Results**: Between 30.12.2021 and 10.09.2024, 22 consecutive patients were prospectively enrolled in the study. CCM implantation was performed for 19 patients at a mean time of 59 ± 65 days. Under active CCM therapy, there was an improvement in GLS, LV-EF, and the Kansas City Heart Failure Questionnaire (KCCQ; all *p* < 0.05). A linear regression analysis showed that the positive effect of CCM on GLS was especially pronounced in patients with a female sex, a non-ischemic etiology of heart failure, and age ≤ 69 years, respectively (all *p* < 0.05). **Conclusions**: CCM therapy is not only linked to an improvement in LV-EF but also increases the global longitudinal strain and quality of life of patients with HFrEF.

## 1. Background

Speckle tracking echocardiography is a non-invasive method for the assessment of left ventricular pump function. Compared to planimetric analysis, strain analysis is a more accurate and, in particular, more examiner-independent method of assessing the contractile movement of the myocardium [1]. During the cardiac cycle, the ejection of blood into circulation is generated via the change in the shortening and relaxation of the heart muscle. The active and passive deformation of the myocardium can be quantified as a strain rate using myocardial deformation analysis. Strain is defined as the deformation of an object in correlation to its original length. The heart, as a three-dimensional object, has three strains along an x-, y-, and z-axes, respectively, and additional strains due to shear forces. During a normal heartbeat, longitudinal, circumferential, and radial shortening can, therefore, be assessed. The extent of this deformation can be specified as a percentage, with a positive value indicating an increase and a negative value indicating a decrease in size. The calculation is based on the following formula: strain = [length (end-systolic) − length (end-diastolic)]/length (end-diastolic). The longitudinal (shortening along the ventricular axis) and circumferential strain (tangential shortening in the cross-section corresponding to a reduction in the ventricular diameter in the short axis) are given a negative sign and the radial strain (thickening of the myocardium during contraction in the short axis) a positive sign. The change in strain per time is referred to as the strain rate and is expressed in units of 1/s.

During strain analysis, so-called speckles (spots) are recognized as reflection patterns, and their extent of movement and deformation is observed and measured. A myocardial segment has several of these speckles, which are stored as a ‘fingerprint’ via specialized software and tracked throughout the cardiac cycle. The software then generates the strain for the respective myocardial segments (regional) and the entire ventricle (global). The conventional LV strain is the difference between the diastolic and systolic states. According to a current study, the LV global longitudinal strain (GLS) in healthy subjects is −22.5 ± 2.7% [2,3]. In addition to GLS, conclusions can also be drawn about the regional limitation of LV contractility. It has been reported that ischemia-related kinetic disturbances and diseases of the left ventricle can thus be detected much more effectively by using strain analysis compared to conventional methods [2,3]. The precise determination of LVEF using echocardiography requires optimal image quality, so blurred images can lead to inaccurate measurements. In addition, the measurements are based on geometric assumptions about the shape of the left ventricle, which is why deviations can affect the accuracy of LVEF determination [4]. Furthermore, LVEF is a load-dependent parameter, which means that changes in preload and afterload may alter LVEF without actual changes in myocardial contractility. In subclinical myocardial damage, LVEF often still shows normal values. Studies have shown that strain analysis is significantly more sensitive to early myocardial dysfunction. The strain can, therefore, detect subclinical myocardial damage even if the LVEF is still normal [5,6,7].

Despite these limitations, the determination of the left ventricular ejection fraction (LVEF) is the most frequently used parameter in transthoracic echocardiography to describe systolic heart function, and it is of utmost importance for the classification of the different phenotypes of heart failure. However, there are several reports suggesting that strain analysis may offer better prognostic relevance. A study from 2018 showed that every 15% increase in GLS (global longitudinal strain) is associated with a 5% reduced mortality risk (hazard ratio [HR]: 0.95; 95% confidence interval [CI]: 0.93 to 0.96; *p* < 0.001) [8].

For patients suffering from heart failure with reduced ejection fraction, lifestyle modification and medical therapy are the cornerstones of treatment [9]. In addition to this, device-based therapies should be implemented to reduce the risk of sudden cardiac death and to improve symptoms and prognosis of the disease (implantable cardioverter defibrillator (ICD); cardiac resynchronization therapy (CRT) if indicated). Additionally, cardiac contractility modulation (CCM) can be used especially in patients with HFrEF who are not candidates for CRT. Based on data from previous studies, CCM therapy is particularly recommended for patients with an LVEF of 35% or less, symptomatic heart failure (NYHA class II/III), and a normal QRS complex despite optimal drug therapy [10,11]. CCM therapy increases the contractility of the heart by directly changing several biochemical processes in cardiomyocytes. The non-excitatory stimulation of the myocardium during the absolute refractory period leads to an increased influx of calcium ions, which, in turn, leads to a prolonged action potential and, in turn, to an improvement in contractility and pump function. Clinical data on the effectiveness of CCM therapy in patients with chronic heart failure have been derived both from randomized trials and from large registry data [12,13,14,15,16,17,18,19,20].

There are several reports showing that CCM therapy leads to an increase in LV ejection fraction for patients with chronic heart failure. Moreover, survival rates at 1 and 3 years were significantly better at 5.2% and 29.5%, respectively, compared to the estimated rates of 18.4% and 40%, based on the MAGGIC risk score [10]. However, to date, no data exist as to whether the application of cardiac contractility modulation also leads to changes in left ventricular strain. Against this background, we conducted a prospective, randomized clinical trial involving patients with chronic heart failure and a reduced ejection fraction to determine whether the application of CCM therapy influences left ventricular global longitudinal strain after 3 months of therapy.

## 2. Methods

We conducted a prospective clinical trial at the Department of Internal Medicine II, Catholic Hospital “St. Johann Nepomuk”, Erfurt, Germany. During the period from 30.12.2021 to 10.09.2024, all patients older than 18 years with impaired left ventricular function, a QRS complex of less than 130 ms, and a state of suffering from chronic heart failure despite guideline-directed medical therapy who planned to undergo CCM implantation were eligible for study inclusion. The study was approved by the Ethics Committee of the Thuringian Medical Association and is registered in the German Register of Clinical Trials (DRKS00027533). The inclusion and exclusion criteria are presented in Table 1.

After written informed consent was obtained, demographic data such as gender, age, height, and weight, as well as chronic comorbidities and current medications, were recorded. Additionally, NT-pro BNP and eGFR were determined for all patients. Each patient underwent a standardized echocardiography assessment conducted by an experienced physician at study inclusion and after 3 and 6 months, respectively. All examinations were performed using a Vivid E95 (GE Healthcare, Chicago, IL, USA) with a focus on a precise and detailed assessment of the left ventricle, including comprehensive strain analysis. Quality of life was assessed using the Kansas City Heart Failure Questionnaire (KCCQ). Patients were scheduled for follow-up visits every three months, at which the status regarding CCM therapy was documented and the echocardiography examination was repeated.

**Statistics:** Statistical analysis was performed using SPSS (version 29). Metric variables are presented as mean values with standard deviations. For comparisons of two groups, normal distribution was first tested using the Shapiro–Wilk test. If a normal distribution was found, Student’s t-test was used; otherwise, the Mann–Whitney U-test was applied. The primary study endpoint was the change in LV global longitudinal strain. In order to analyze the impact of several variables on the treatment effect of CCM therapy regarding LV global longitudinal strain, a linear regression model was used (exploratory analysis).

## 3. Results

Between 30.12.2021 and 10.09.2024, 22 patients were included in the study. The baseline parameters of these patients are presented in Table 2.

**Figure 1:**
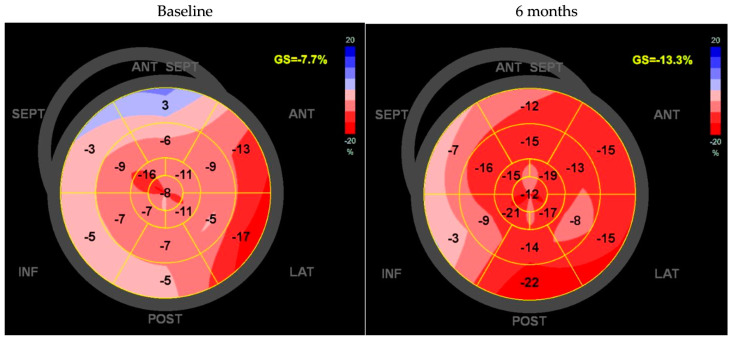
Example of LV strain analysis for a patient at the baseline and after 6 months of CCM therapy.

During the follow-up period, CCM implantation was performed for 19 patients at a mean time of 59 ± 65 days after the baseline. After implantation, CCM therapy was delivered for 6.3 ± 2.3 h per day (standard setting: 1 h “on”, followed by 2.25 h “off”; voltage: 7.5 V/duration: 20.5 ms). The effects of CCM therapy on the echocardiographic parameters of the left ventricle are presented in Table 3. As can be seen from the table, it turned out that, under active CCM therapy, there were improved values for GLS, LV-EF, and KCCQ, respectively. Figure 1 provides an example of the changes in LV strain for a representative patient.

**Figure 2:**
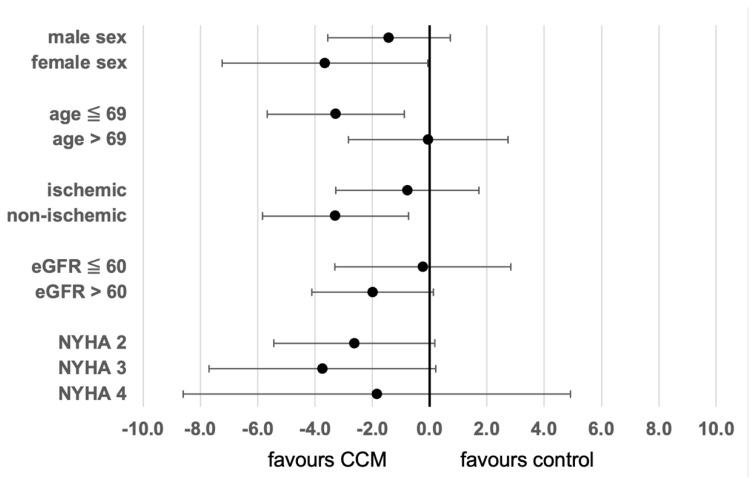
Size of effect of CCM therapy on LV global longitudinal strain in different subgroups of patients (regression coefficient with 95% confidence intervals).

In order to analyze the impact of several variables on the treatment effect of CCM therapy regarding LV global longitudinal strain, a linear regression model was used. Figure 2 shows a forest plot depicting the effect size of CCM therapy on GLS in several patient subgroups. As can be seen from the graph, the effect of CCM therapy on GLS was especially pronounced for female patients, patients ≤ 69 years of age, and patients with a non-ischemic etiology of heart failure, respectively. However, in a multivariate model including the variables CCM therapy, sex, etiology of heart failure, age, and eGFR, only CCM therapy was significantly correlated with LV GLS.

### Safety Evaluation

The frequency of adverse events during follow-up is shown in Table 4. Neither death, myocardial infarction, nor stroke were documented for any patient, and no adverse event related to ICD cross-talk was observed. There was one sudden cardiac arrest of a patient with non-ischemic cardiomyopathy who had no previous ICD implant.

## 4. Discussion

This study was performed to investigate whether the implementation of CCM therapy in patients with systolic heart failure would lead to a change in LV function and especially in left ventricular global longitudinal strain. Previous studies had already shown a significant improvement in left ventricular ejection fraction after CCM implantation, which could be confirmed through the results of our study [10,15]. Additionally, our data show that left ventricular global longitudinal strain is also improved via CCM. In parallel with the capture of our data, a monocentric observational study was published demonstrating that there is an increase in GLS after CCM implantation [21]. However, the cited study described neither the detailed effects of CCM on GLS in special patient subgroups nor the effects on the quality of life of the patients.

Changes in GLS among patients with chronic heart failure are of special interest, as it has been reported that GLS is closely linked to the prognosis of patients suffering from HFrEF [8]. In addition, long-term studies show that, during an average follow-up period of 34.2 months after CCM implantation, mortality rates at 1 and 3 years of 5.2% and 29.5%, respectively, were significantly better than the rates estimated using the MAGGIC risk score of 18.4% and 40% [10]. It has also been reported that CCM may be an effective therapy for patients with severe heart failure after heart transplantation, especially when conventional treatments are not sufficiently effective [22].

Cardiac resynchronization therapy (CRT) has been shown to improve clinical outcomes in patients with HFrEF and a wide QRS complex in a number of clinical studies and is nowadays an indispensable treatment option for these patients. In parallel, CRT has also been shown to improve global longitudinal strain (GLS) in several studies [23,24] as well as parameters of RV function [25]. Likewise, in data from MADIT-CRT patients with left ventricular dyssynchrony, non-ischemic cardiomyopathy, and wide QRS complexes who were treated with CRT-D exhibited significantly greater improvements in GLS than those in the ICD group (CRT-D: −1.4 ± 3.1%; ICD: −0.4 ± 2.5%; *p* < 0.001). Additionally, each 1% improvement in GLS within one year was associated with a 24% reduction in the primary endpoint of all-cause death or non-fatal heart failure events (*p* < 0.001) [24]. It is, therefore, important to note that our study now shows that CCM therapy also improves GLS, which, in turn, should contribute to the clinical improvements that have been described for CCM therapy before [13,15,18,26].

The present study demonstrates that CCM leads both to subjective and functional improvements for patients with reduced left ventricular ejection fraction. This can be seen in an improvement in subjective health status as measured using the KCCQ questionnaire. Similar results have already been observed in previous studies investigating the effect of CCM therapy on NYHA stages. In the FIX-HF-5 study, for example, a significant improvement in the Minnesota Living with Heart Failure Questionnaire (MLWHFQ) was observed after six months of CCM therapy compared to the control group [20]. Additionally, a pilot study of CCM for patients with HFpEF also reported an improvement in the KCCQ score with an increase of 18.0 ± 16.6 points [27]. This value was even higher in our investigation for patients with HFpEF. In general, an increase in the KCCQ score by 20 points is considered a substantial improvement in quality of life, and an increase by 5 or more points is independently associated with reduced mortality and heart failure hospitalization, demonstrating greater sensitivity to meaningful clinical changes compared to NYHA classes over time [28]. However, due to the open-label design, it should be taken into consideration that our results might have been influenced by the placebo effect to a certain degree.

**Conclusions**: Our data indicate that CCM therapy is not only linked to an improvement in LV-EF but also increases global longitudinal strain and quality of life among patients with HFrEF. The findings suggest that especially patients with a female sex, a non-ischemic etiology of heart failure, and age ≤ 69 years might experience the greatest benefit of CCM therapy in this regard.

## 5. Limitations

Due to the limited number of patients, our study was not intended to provide statistical information about the effects of CCM on the frequency of clinical endpoints like heart failure hospitalizations or mortality. Additionally, the results might have been influenced by placebo effects due to the open-label design of the study. The findings from the regression analysis especially have to be considered hypothesis-generating, and they require confirmation with larger cohorts.

## Figures and Tables

**Table 1:** Inclusion and exclusion criteria.

Inclusion Criteria	Exclusion Criteria
Age < 18 years	Planned interventional or surgical procedures other than CCM implantation within the next 3 months
Chronic heart failure NYHA II, III, or IV	Inability to assess heart function via echocardiography
LVEF 25–45%	Lack of consent to participate in the study
Guideline-based pharmacological treatment	Inability to attend follow-up visits
Clinical indication for CCM therapy	
QRS complex < 130 ms	
Written informed consent	

**Table 2:** Demographic parameters, estimated glomerular filtration rate (GFR), NT-proBNP, and medical history at baseline.

Parameter	Mean (All Patients) [N = 22]
**Male sex**	16 (73%)
**Age [years]**	70 ± 6
**Height [cm]**	170 ± 10
**Weight [kg]**	96 ± 22
**GFR [mL/min∗1.73 m^2^]**	62 ± 17
**NT-pro BP [pg/mL]**	2669 ± 3716
**Etiology of heart failure**	
**Ischemic cardiomyopathy**	11 (50%)
**Non-ischemic cardiomyopathy**	11 (50%)
**NYHA stage**	
**NYHA I**	0 (0%)
**NYHA II**	4 (18%)
**NYHA III**	12 (55%)
**NYHA IV**	6 (27%)
**Hypertension**	18 (82%)
**Diabetes mellitus**	14 (64%)
**Coronary heart disease**	11 (50%)
**History of PCI**	11 (50%)
**History of CABG**	1 (5%)
**History of heart value surgery**	6 (27%)
**Antiplatelet therapy (APT)**	
**Single APT**	9 (41%)
**Dual APT**	2 (9%)
**None**	11 (50%)
**(D)OAC**	11 (50%)
**ß-blockers**	19 (86%)
**ACE inhibitors**	3 (14%)
**ARB**	1 (5%)
**Sacubitril/Valsartan**	18 (82%)
**MRA**	15 (68%)
**SGLT2 inhibitors**	14 (64%)
**Diuretic** **LVDd [mm]** **LVDs [mm]** **LVVd [mL]** **LVVs [mL]** **LV-EF [%]** **Global longitudinal strain [%]** **KCCQ [points]**	19 (82%) 58.3 ± 8.6 46.6 ± 9.8 167.7 ± 54.8 107.1 ± 48.2 34.4 ± 8.1 −9.2 ± 3.2 31 ± 16.8

**Table 3:** Parameters of transthoracic echocardiography and quality of life, depending on the delivered therapy (active CCM or no active CCM).

Parameter	Active CCM (N = 22)	No Active CCM (N = 39)	*p*-Value
**LVDd [mm]**	56.1 ± 11.0	60.4 ± 11.0	0.193
**LVDs [mm]**	45.9 ± 13.6	49.3 ± 11.3	0.592
**LVVd [mL]**	172.4 ± 69.2	174.7 ± 61.6	0.859
**LVVs [mL]**	99.2 ± 59.4	111.7 ± 46.9	0.377
**LV-EF [%]**	41.7 ± 10.1	35.5 ± 7.6	<0.05
**GLS [%]**	−10.3 ± 3.7	−8.3 ± 3.2	<0.05
**KCCQ [points]**	62.0 ± 21.3	35.1 ± 20.1	<0.05

**Table 4:** Adverse events and serious adverse events at 6 months after the baseline.

Parameter	Value (N = 22)
**ACS**	0 (0%)
**Stroke**	0 (0%)
**Hospitalization for heart failure**	6 (27%)
**Cardiac arrest**	1 (4%)
**Hospitalization for other causes**	8 (36%)
**Cardiovascular procedures**	5 (23%)
**Death**	0 (0%)

## Data Availability

Data can be obtained from the authors upon reasonable request.
